# The Breeding Season and Movement Ecology of Male White‐Tailed Deer in Southwest Wisconsin

**DOI:** 10.1002/ece3.71589

**Published:** 2025-07-09

**Authors:** Matthew A. Hunsaker, Marie L. J. Gilbertson, Daniel J. Storm, Wendy C. Turner

**Affiliations:** ^1^ Wisconsin Cooperative Wildlife Research Unit, Department of Forest and Wildlife Ecology University of Wisconsin–Madison Madison Wisconsin USA; ^2^ Wisconsin Department of Natural Resources Eau Claire Wisconsin USA; ^3^ U.S. Geological Survey, Wisconsin Cooperative Wildlife Research Unit, Department of Forest and Wildlife Ecology University of Wisconsin–Madison Madison Wisconsin USA

**Keywords:** changepoint analysis, chronic wasting disease, hunting, *Odocoileus virginianus*, reproductive behavior, rut, white‐tailed deer

## Abstract

White‐tailed deer ( *Odocoileus virginianus* ; hereafter, deer) have been widely studied regarding their breeding ecology and responses to hunting pressures. However, variations in defining the breeding season—its duration and timing—across studies have created uncertainty about whether regional differences in deer breeding ecology stem from ecological factors or methodological inconsistencies. This study aims to clarify the peak breeding season timing and the movement patterns of males during this period, particularly in relation to hunting seasons. Understanding how age and the timing of hunting seasons impact movement and breeding behaviors is important for wildlife managers, as these factors can affect harvest success. This study took place in southwest Wisconsin, using GPS data collected from 188 collared male deer between 15 October and 1 December from 2017 to 2020. Based on generalized linear mixed models, 2‐year‐old males exhibited higher hourly movement rates than other ages, and the opening weekend of the firearm hunting season had no significant effect on movement rates. In contrast, the variance in daily movement rate differed significantly between yearlings and older ages, with males 3 years and older displaying the highest variance. This suggests that older males may alternate more frequently between high‐movement mate searching and lower‐movement mate tending, potentially enhancing reproductive success. Similarly, 2‐year‐old males had larger daily ranges than both older and younger ages. Changepoint analysis of daily movement rates determined that the peak breeding season occurred between 23 October and 12 November, with little variation among ages and alternative metrics. Our findings indicate that male movement rates and ranges can reflect deer reproductive efforts and vary by age, which has important implications for reproductive success and disease transmission risk.

## Graphical abstract

This study investigates the peak breeding season and movement patterns of white‐tailed deer (WTD) in southwestern Wisconsin using GPS data from 188 collared males. Results indicate that two‐year‐olds have higher movement rates, while older males exhibit greater variability, suggesting reproductive advantages. The peak breeding season is identified as October 23 to November 12, highlighting the relevance of male movement patterns in disease risk and wildlife management decisions.

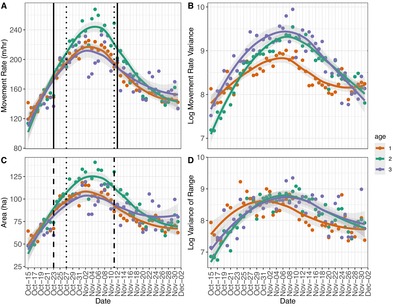

## 1 Introduction

Sexual selection theory suggests that size dimorphism between males and females, male secondary sexual characteristics (e.g., antlers, horns), body condition, and aggressiveness evolved for males to gain reproductive opportunities in polygynous species (Geist 1966; Andersson 1994; Clutton‐Brock et al. 1988; Hoem et al. 2007). Mating strategies for mammalian males are influenced by female spatial distribution and reproductive synchrony (Emlen and Oring 1977; Clutton‐Brock 1989). Females in estrus may be widely distributed in small groups, exhibit unpredictable timing of estrus, and be attended by a small subset of males or unattended altogether (Wauters et al. 1990; Ryser 1992), leading to scramble competition where all males have potential access to mates (Schwagmeyer 1988).

Within scramble competition, various strategies may be used by males for mate searching, depending on the presence of other competing males and the probability of encountering a female (Apollonio et al. 1992; Hogg and Forbes 1997; Isvaran 2005). White‐tailed deer ( *Odocoileus virginianus* ; hereafter, deer) are one important example that demonstrates heterogeneity in mate‐search strategy. The deer mating system is characterized by polygyny, sexual dimorphism, reproductive synchrony, and age‐based differences in male mating success. Mating in white‐tailed deer is representative of scramble competition because males aged ≥ 3 years were responsible for the majority of offspring in multiple studies, but younger males between 1.5 and 3 years still bred successfully (Sorin 2004; DeYoung et al. 2009; Turner et al. 2016).

Breeding behavior in male *Odocoileus* spp. is detectable through a variety of changes in movement behavior during the rut. Males often increase home range size and movement rates during the rut to search for receptive females (Nelson and Mech 1981; Kammermeyer and Marchinton 1977; Marchinton and Hirth 1984). When a receptive female is found, males will attempt to tend the female for a roughly 24‐h period while the female is in estrus (Hamilton et al. 1985; Haugen 1959; Verme 1965; Knox et al. 1988). This behavioral pattern means that reproductively successful males are likely switching between a searching state, characterized by high movement rates, and a tending state, characterized by low movement rates. However, as with mate search strategies, these movement patterns are expected to vary by age. Adults are typically more successful at breeding and likely display a roving search strategy, characterized by males traveling between known groups of females (Whitehead 1990). In contrast, yearlings likely display a resident or Levy search strategy, wherein males remain proximate to one group of females or range more widely in search of accessible female groups, respectively (Whitehead 1990; Sorin 2004; DeYoung et al. 2009; Turner et al. 2016; Viswanathan et al. 2000; Bartumeus et al. 2002; Humphries et al. 2010).

Variation in mating strategies in males by age or condition can result in heterogeneity in space use and subsequent inter or intrasex contact patterns. Such heterogeneity can have consequences for other ecological and management processes such as infectious disease transmission and hunter harvest success. For example, hypotheses to explain increased prevalence of chronic wasting disease (CWD) in males include that males have high contact rates with infectious females during the breeding season or rut (hereafter, rut), contact rates within bachelor groups are higher than contact rates between matriarchal groups, and that males have larger home ranges—and thus increased environmental risk of infection—than females (Samuel and Storm 2016; Potapov et al. 2013; Silbernagel et al. 2011; Grear et al. 2006; Saunders et al. 2012; Kelly et al. 2010; Ketz et al. 2019). Thus, understanding heterogeneity in male breeding behavior and movements may inform deer population management and disease control and surveillance efforts (Evans et al. 2016; Edmunds et al. 2018). Our study aims to determine how male movement behavior during the rut varies by age and external factors such as hunting seasons and weather.

In addition, recreational hunting is the main mechanism for ungulate population and disease management across much of North America, and disturbance from hunting activity could influence deer movement patterns, including during the rut. Animal responses to human predation risk will vary depending on factors such as type of risk, environment, and temporal scale (Dasmann and Taber 1956; Van Etten et al. 1965; Kammermeyer and Marchinton 1976; Kilgo et al. 1998; Karns et al. 2012). In deer, it may be difficult to differentiate responses to hunting pressure versus typical rutting behavior due to variability in hunting pressure and the timing of the hunting season in relation to the rut (Tomberlin 2007; Karns et al. 2012; Little et al. 2016). For example, in Wisconsin, the 9‐day firearm season always begins on the Saturday in November just prior to the U.S. Thanksgiving holiday, such that, across years, the onset of this primary recreational hunting period ranges from 17 to 23 November. When opening day of the firearm hunting season falls earlier in the year, it occurs closer to the peak rut, and may therefore be expected to motivate higher magnitude responses in deer. These responses may take the form of decreased movement rates, due to an avoidant response to perceived risk (Nixon et al. 1991; Vercauteren and Hygnstrom 1998; Kilpatrick and Lima 1999); alternatively, deer may increase movement rates as human activity disturbs them from typical resting or breeding behaviors (Van Etten et al. 1965; Marshall and Whittington 1968; Root et al. 1988; Naugle et al. 1997). Increased deer movement is thought to increase vulnerability to harvest (Little et al. 2016), so understanding how the timing of the hunting season, in combination with deer responses to harvest pressure, affects movement could influence wildlife managers' decisions on timing of hunting seasons to accomplish population and disease management goals.

White‐tailed deer have a broad geographical range in North America, such that reproductive movement patterns in male deer likely vary by geographic region. However, such relationships are obscured across studies by inconsistent determination of the timing of the deer rut. While the majority of female deer in temperate populations are bred in a 2–4 week period (DeYoung and Miller 2011; Hewitt 2011), the deer rut has been defined variably as: “breeding season” from October 1 through December 15 in Illinois (Nixon et al. 1991), October 10–December 31 in Wisconsin (Skuldt et al. 2008), “rut” from October 21–December 7 in Virginia (Hölzenbein and Schwede 1989), November 18–December 1 in Oklahoma (Webb et al. 2009), and “peak rut” as December 2–15 in Texas (Foley et al. 2015). The rut varies latitudinally (Bubenik et al. 1990), so standardizing methods to identify the rut or the peak rut will allow studies to define these periods based on regionally appropriate, ecologically‐relevant data, and make appropriate comparisons between other regions or studies.

The objectives of this study were to characterize male deer movement ecology during the rut, under the presumption that movement metrics during this time period are related to breeding behavior, by (1) defining the peak rut using relevant movement metrics such as movement rates and daily short‐term occupancy distributions (hereafter, ranges) and neonate conception data, (2) evaluating differences in movement behavior and how these varied by age, and (3) identifying the main drivers of movement rates during the rut, including determining how the timing of the major firearm hunting season affects male movement. We expected all males to increase movement rates and range sizes up to the peak rut, followed by a slow decline (Nelson and Mech 1981; Kammermeyer and Marchinton 1977; Marchinton and Hirth 1984; Hellickson et al. 2008; Webb et al. 2009). We further expected yearling males to have the lowest movement rates during the rut because they are not fully sexually mature, resulting in inexperience and the inability to defend mates from more mature males (Foley et al. 2015). We propose that within‐individual variance in movement rates may be a useful indicator of breeding success during the rut as males switch between higher movement rate searching and lower movement rate tending. Yearling males were therefore expected to display the lowest variance in movement rate, as they are least likely to breed successfully (Sorin 2004; DeYoung et al. 2009; Turner et al. 2016). Results of our study provide insight into variation in male mating behavior, with potential relevance to wildlife management.

## 2 Materials and Methods

### 2.1 Study Area

**FIGURE 1:**
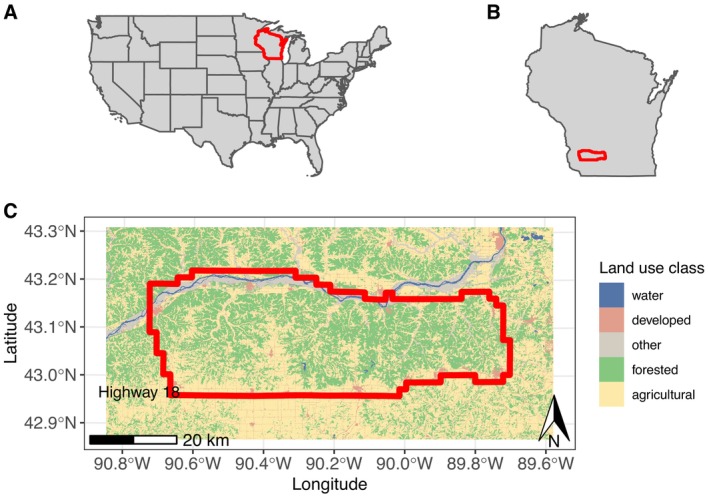
Maps showing (A) the state of Wisconsin (red polygon) within the continental United States, (B) the study area (red polygon) in southwest Wisconsin, and (C) the study area (red polygon) with the landscape colored by land use class. In (B), the red box corresponds to the red polygon of the area shown in (C). Map colors correspond to National Land Cover Database (NLCD) land use classifications (Dewitz 2021); of the 5.2% of land pictured classified as “other,” about 84.5% is woody or emergent herbaceous wetlands, with the remainder a mix of barren land, shrub/scrub, and grassland/herbaceous. The southern border of the study area is Highway 18. The major river pictured along the northern border is the Wisconsin River.

The Wisconsin Department of Natural Resources (WDNR) trapped and collared deer in southwest Wisconsin from December 2016 to March 2020, in portions of Dane, Iowa, and Grant counties. This area is largely under private ownership. Land cover in the study area is a mixture of mostly deciduous forest and agriculture (Figure 1). The landscape consisted of forested hills dominated mainly by hardwoods, valleys of warm and cool season grasslands, and agriculture comprising a mix of row crops, hay fields, and pasture (primarily cool season grasses). Agricultural crops were primarily corn ( *Zea mays* ), soybean ( *Glycine max* ), and alfalfa ( *Medicago sativa* ). From 2017 to 2020, the mean extreme maximum temperature was 33.2°C, the mean extreme minimum was −28.1°C, and the mean annual temperature was 7.7°C. Average annual rainfall during the study was 117.7 cm, and average snowfall from 2018 to 2020 was 121.5 cm (NCEI 2021).

The timing of the WDNR‐managed deer hunting seasons varies by weapon type. Archery season begins the second Saturday in September and ends in January. The firearm deer season starts the third Saturday of November and lasts 9 days. This is followed directly after by a muzzleloader season for another 9 days. The firearm deer seasons during this study were from November 18–26 in 2017, November 17–25 in 2018, November 23–December 1 in 2019, and November 21–29 in 2020 (Table S1).

### 2.2 Deer Capture

Adult deer were captured and fitted with global positioning system (GPS) collars (Vectronic VERTEX Lite Iridium, Vectronic Aerospace, Germany or Lotek LiteTrack Iridium 420, Lotek Wireless, Canada) from December through March starting in December 2016 through March 2020 (4 winters, 2017–2020). Deer were trapped using modified clover traps, Stephenson box traps (Hawkins et al. 1967), and drop nets (Ramsey 1968). Once captured, individuals were chemically immobilized with intramuscular injections of 27.3 mg/mL butorphanol+9.1 mg/mL azaperone+10.9 mg/mL medetomidine (BAM; Miller et al. 2009) via hand‐injection. Individuals were monitored via rectal temperature, respiratory and heart rates, and capillary refill time. Deer age was recorded as fawns (< 1 year), yearlings (≥ 1 year, < 2 years), and adults (≥ 2 years) based on tooth emergence, wear, and replacement (Severinghaus 1949) and the incisiform canine was collected from adults for more accurate aging (Gilbert 1966). During the 2017 and 2018 capture seasons, yearling and older females were fitted with a vaginal‐implant transmitter (VIT; Sirtrack P02949). Following capture procedures, deer were reversed from chemical immobilization with atipamezole (25 mg/mL). All deer capture and handling protocols followed American Society of Mammalogists guidelines (Sikes et al. 2011) and were approved under WDNR's Animal Care and Use Committee (Protocol: 16‐Storm‐01).

Location information was collected from GPS collars hourly during the rut for males, but otherwise was collected every 4 h, and sometimes as infrequently as every 13 h to conserve battery life. The high‐intensity hourly sampling period for males is the focal time period for this study, occurring between 15 October and 1 December. For females fitted with vaginal‐implant transmitters, these were monitored daily the following May through June (2017–2018) to locate and capture neonatal fawns. A pulse rate change for a VIT frequency indicated that it had been expelled and field crews located transmitters via very high frequency (VHF) telemetry. The area around the expelled VIT was then searched for fawns (Carstensen et al. 2003). Additionally, fawns not associated with a VIT were captured by conducting foot searches (Steigers and Flinders 1980; Ballard et al. 1999; Vreeland 2002), by identifying females displaying postpartum behavior, and examining GPS location clusters of collared adult females (Downing and McGinnes 1969; White et al. 1972). All neonatal fawns were captured during the months of May and June, 2017–2019. Neonate capture data were used to estimate the timing of conception by backdating 200 days from birth to reflect gestation period (DeYoung et al. 2002). Birth dates of neonatal fawns were estimated using umbilicus characteristics described by Haugen and Speake (1958). Umbilicus characteristics were described as, “fresh bloody,” “moist,” “present but dry,” “absent but scabbed over,” or “absence of scab” at capture. To more accurately describe birth dates in relation to capture dates, a “fresh bloody” umbilicus indicated day of birth, “moist” and “present but dry” was 1 day old, “absent but scabbed over” was 2 days old, and “absence of scab” was not used due to uncertainty in age (Haugen and Speake 1958).

### 2.3 GPS Collar Data Processing

Prior to analysis, deer GPS collar data were screened for likely erroneous locations. The GPS collar data processing is described in Gilbertson et al. (2022). In brief, locations were removed based on dilution of precision values and improbable “spike” movements (i.e., rapid out‐and‐back movements; Lewis et al. 2007; Bjørneraas et al. 2010). Following processing, we included only males with movements during the high intensity sampling period (October 15–December 1). Individual dispersal events identified from Gilbertson et al. (2022) were removed from the data set, as movements of these deer are not necessarily related to mate searching behavior and would bias estimates of movement rates and range sizes. To further screen points, we removed specific dates associated with unique individuals that had a mean fix rate slower than 2.5 h or that had fewer than 19 fixes per individual. This ensured consistent fine‐scale temporal GPS collar data across individuals.

### 2.4 Statistical Analysis

#### 2.4.1 Daily Age‐Summarized Movement Patterns

To identify drivers of movement patterns in deer during the rut, we fit polynomial regression models to daily summary statistics for movement rates and range sizes. We calculated movement rates (m/h) for each individual as the distance between sequential locations (i.e., step length) divided by the time elapsed to standardize the measurements. The minimum and maximum time elapsed between consecutive points was 0.5 and 2.4 h respectively, with a mean elapsed time of 1.0 h (Table S1). We estimated daily range sizes (ha) for each individual using Brownian bridge movement modeling (BBMM) in the *adehabitatHR* package in R (v4.0.5; Calenge 2006; R Core Team 2021). We obtained daily 95% utilization distributions (UD) for each individual on each day of each year.

We calculated summary statistics of movement rates and range size (mean, ${\underline{x}}_{\mathrm{ij}}$ and variance, ${s^{2}}_{\mathrm{ij}}$, for the i'th age‐class and j'th day) across all individuals on a given day (1–48 corresponding to October 15–December 1; d) and of a given age‐class (1.5, 2.5, 3.5+; a) calculated as:

$${\bar{X}}_{\mathrm{ij}}=\frac{1}{a\cdot d}\sum_{i=1}^{a}\sum_{j=1}^{d}x_{\mathrm{ij}}\;\;{s^{2}}_{\mathrm{ij}}=\frac{1}{\left(a\cdot d\right)-1}{\left(\left(\sum_{i=1}^{a}\sum_{j=1}^{d}\right)-{\bar{x}}_{\mathrm{ij}}\right)}^{2}$$

We then fit polynomial regression models predicting daily mean movement rate (in m/h; m), daily mean range size (in ha; r), log daily movement rate variance, and log daily range variance. Log transformations were used to satisfy assumptions of normality. Model covariates included (1) day of the year, treated as a continuous polynomial variable; (2) age, treated as a categorical variable; and (3) the interaction between the day number and age (see Appendix S1, for more details on polynomial regression equations). We used a forward selection process and Akaike information criterion (AIC) to choose a top polynomial regression model for the day number covariate (Burnham and Anderson 2004; Johnson and Omland 2004).

#### 2.4.2 Daily Individual Movement Patterns

We examined the effects of age and hunting on movement rates by fitting a linear mixed‐effects model. We calculated mean movement rates for each unique individual deer (z), on day number (*d*), and of age (*a*), such that the mean movement rate for the *i*'th individual on the *j*'th day of the *k*'th age was calculated as:

$${\bar{X}}_{\mathrm{ijk}}=\frac{1}{z\cdot a\cdot d}\sum_{i=1}^{z}\sum_{j=1}^{a}\sum_{k=1}^{d}x_{\mathrm{ijk}}$$

We evaluated the square root of individual mean daily movement rate as a function of day number, age, hunting season (archery, opening weekend of firearm, and firearm; firearm includes firearm season and start of muzzleloader season through 1 December; h), and temperature (t), with a random effect for individual deer (A). Weather data were obtained from the National Centers for Environmental Information (NCEI 2021), from a weather station located within the study area in southwest Wisconsin. The weather variables we considered were the daily minimum and maximum temperatures (°C). As in the age‐summarized movement pattern models, day number was modeled as a continuous polynomial variable, and we evaluated an interaction between day number and age:

$$\begin{array}{cc} \sqrt{m_{\mathrm{ij}}}\;=\; & \beta_{0}+\beta_{1}d_{1i}+\beta_{2}{d^{2}}_{2i}+\beta_{3}{d^{3}}_{3i}+\beta_{4}{d^{4}}_{4i}+\beta_{5}{d^{5}}_{5i}\\ & +\beta_{6}{d^{6}}_{6i}+\beta_{7}a_{1i}+\beta_{8}{\left(\mathrm{ad}\right)}_{1i}+\beta_{9}{\left(\mathrm{ad}\right)}_{2i}^{2}\\ & +\beta_{10}t_{1i}+\beta_{11}h_{1i}+\beta_{12}{\left(\mathrm{hd}\right)}_{1i}+A_{i}+\varepsilon_{\mathrm{ij}} \end{array}$$

$$A_{i}\sim N\left(0,{\theta^{2}}_{A}\right)$$

$$\;\varepsilon_{\mathrm{ij}}\sim N\left(0,\theta^{2}\varepsilon \right)$$

We used square root transformation of movement rate to satisfy model assumptions of homoscedasticity and normality. We fit four candidate models and used AIC to determine the top model. In the first three models, the temperature covariate was either the daily minimum, maximum, or change in daily temperature (max‐min). In the fourth model, we dropped the temperature and hunting covariates and tested whether a more general year covariate could better represent the effects of annual variations in temperature, timing of hunting season, or other unmeasured factors. To understand the relative importance of between—versus within‐individual variation in daily mean movement rates, we compared the individual‐level (deer) variance ($A_{i}$) and the residual variance ($\;\varepsilon_{\mathrm{ij}}$) from the top fitted model. The partitioning of these variances reflects the relative contribution of individual‐level and observational‐level variation to overall movement rate variability.

All statistical analyses were conducted using R v3.6.3 and 4.1.2 (R Core Team 2021). We examined plots of residuals and quantile‐quantile plots to evaluate normality and homoscedasticity of variance assumptions.

#### 2.4.3 Daily Activity Patterns

Daily activity patterns (i.e., movements on an hourly basis) were investigated to evaluate if the timing of peak movement or activity changed across the study period. Hourly distance traveled was found by calculating the distance between sequential locations for individual deer. A mean distance (${\underline{d}}_{\mathrm{ijk}}$ for the *i*'th age‐class, *j*'th week, and *k*'th hour) traveled by hour (0–23; h) was then calculated by age (a) and week (w; resulting in, for example, one mean distance traveled estimate for all the yearlings in week 1 and hour 1):

$${\bar{d}}_{\mathrm{ijk}}=\frac{1}{a\cdot w\cdot h}\sum_{i=1}^{a}\sum_{j=1}^{w}\sum_{k=1}^{h}x_{\mathrm{ijk}}$$

Weeks were distinct 7‐day periods within the study period, with week one being October 15–October 21, and so on. We fit a linear regression predicting mean hourly movement rate. Covariates included categorical variables of age, period of day (night, dawn, day, dusk), and an ordered variable, week number, with the age variable interacting with both temporal variables. Period of day was defined for every GPS location and time collected using the *suncalc* package in R (Thieurmel and Elmarhraoui 2019). Dawn was defined as hours after night end and before sunrise, resulting in dawn being the hour of and hour prior to sunrise, while dusk was defined as hours after sunset and before night, resulting in dusk being the hour of and hour after sunset. Day hours were those after sunrise and before sunset and night hours were those after night and before night end.

#### 2.4.4 Timing of the Peak Rut

To define the peak activity of the rut, we conducted changepoint analyses for daily mean movement rates and ranges. We used the *changepoint* package in R (Killick and Eckley 2014; Killick et al. 2022), using the segment neighborhoods method (“SegNeigh”), with AIC as the penalty term and two changepoints (*Q* = 3). We thereby estimated start and end dates for the peak rut, repeating the changepoint analysis for both movement metrics (daily mean movement rates and ranges) for each age separately, and for all males together. To determine if movement‐based estimates for the timing of the peak rut corresponded with the timing of successful breeding events, we performed an additional changepoint analysis for estimated conception events by day. Conception dates were derived from the previously described fawn captures and backdated birth dates.

## 3 Results

We captured 1157 individual deer of which 763 (452 female, 311 male) were > 8 months old at capture and 347 (179 female, 166 male, 2 unidentified) were neonatal fawns. We evaluated movement data during the rut for 188 males that met data requirements for inclusion in the analyses. Thirty‐four of these individuals were monitored for more than 1 year, resulting in movement data for 39 males in 2017, 54 in 2018, 65 in 2019, and 74 in 2020 (Table S1). Age classes included 104 yearlings, 64 two‐year olds, and 64 deer 3 years of age or older (hereafter, 3+; Figure S1). Capture dates from 307 out of 347 neonatal fawns were used for estimates of conception dates.

### 3.1 Timing of the Peak Rut

**FIGURE 2:**
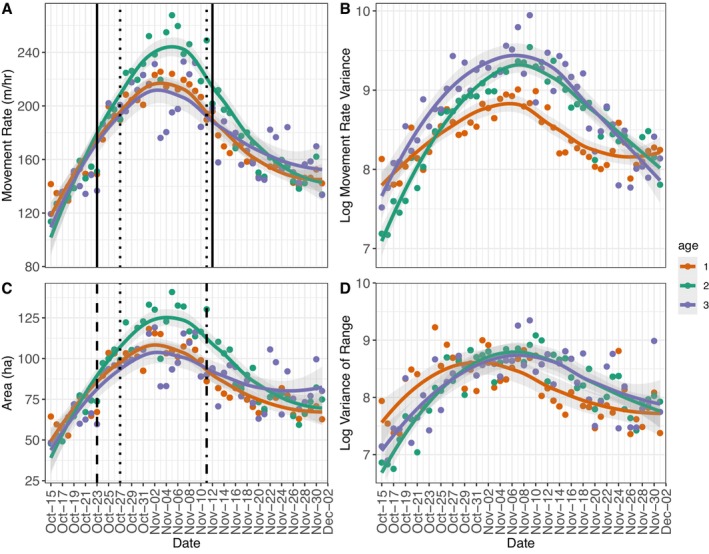
Average daily movement rates (m/h) (A), log variances of daily movement rates (B), range sizes (daily 95% Utilization Distribution) (C), and log variances of range sizes (D) by age (1, 2, 3+) of male white‐tailed deer ( *Odocoileus virginianus* ) during the breeding season in southwest Wisconsin during 2017–2020. Shaded areas represent 95% confidence intervals. Vertical lines represent dates determined from changepoint analysis of the breeding season, based on daily mean movement rate (solid), daily mean range size (dashed) and estimated conception dates backdated 200 days before birth dates established from neonate captures (dotted).

The changepoint analyses to define the peak rut from changes in movement rates, range sizes, or conception dates yielded similar results (Figure 2, Table S2). The start date of the peak rut (range of October 23–27 across analyses) showed similar synchrony across datasets and ages compared to the end date (range of November 9–15 across analyses; Table S2). Peak rut length based on summary movement metrics was 20–21 days long, while from conception dates it was 16 days long.

### 3.2 Daily Age‐Summarized Movement Patterns

Across all statistical analyses of movement metrics, male deer activity tended to increase to a peak in early November, followed by a decrease for the remainder of the study period, with notable differences between ages. When assessing mean daily summarized movement rates and range sizes over the study period (Figure 2), 2‐year‐olds exhibited higher mean daily movement rates and larger mean daily ranges than yearlings and 3+; mean daily movement rates and mean daily range size did not differ between yearlings and 3+ year‐old males (pairwise comparisons; *p* < 0.0001; see Appendix S1 for polynomial regression results; Table S3; Table S4).

All ages showed a trend for an increase followed by a decrease in the variance in log movement rate, with movement variance peaking around 7 November. In contrast to movement rates and range sizes, yearlings showed lower log movement rate variance than the 2‐year olds and 3+ (pairwise comparisons; *p* < 0.0001). The 2‐year olds showed some evidence of lower log movement rate variances than 3+, but this did not reach statistical significance (pairwise comparison; *p* = 0.06; Table S5). In addition, we found that the 2‐year olds had increased log range size variance, as compared to the yearlings (pairwise comparison; *p* = 0.01). The yearlings showed some evidence of lower variance in log range size than 3+, but this did not achieve statistical significance (pairwise comparison; *p* = 0.08), and there was no evidence that the older adult age classes (2‐year olds and 3+) were different from one another (pairwise comparison; *p* = 0.67; Figure 2; see Appendix S1 for polynomial regression results; Table S6).

### 3.3 Daily Individual Movement Patterns

**FIGURE 3:**
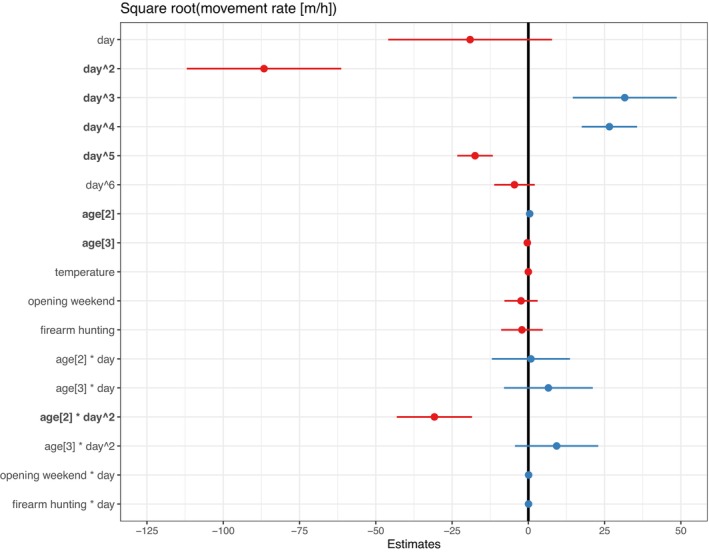
Model results for polynomial mixed model of square root transformed movement rate of male white‐tailed deer ( *Odocoileus virginianus* ) in southwest Wisconsin. Coefficient estimates are shown with 95% confidence intervals. Red results show negative coefficient estimates and blue show positive coefficient estimates. Covariates were statistically significant (bold) if the 95% confidence interval did not cross the vertical black line (estimate = 0). The day^x^ parameter describes the day number covariate raised to the power of x.

Similarly, when evaluating individual‐level mean daily movement rates, the top model found a significant relationship between movement rate and age, in addition to day number (Figure 3; Table S7; Figure S2). As in the summarized model, movement rates had an increasing relationship with day number followed by a decrease. The 2‐year‐olds had significantly higher movement rates than the other age classes (pairwise comparison; *p* < 0.001), while the yearlings did show evidence of higher movement rates than 3+ (*p* < 0.05). The top model did not identify a significant effect of weather, year, hunting seasons, or the timing of opening firearm weekend on movement metrics.

**FIGURE 4:**
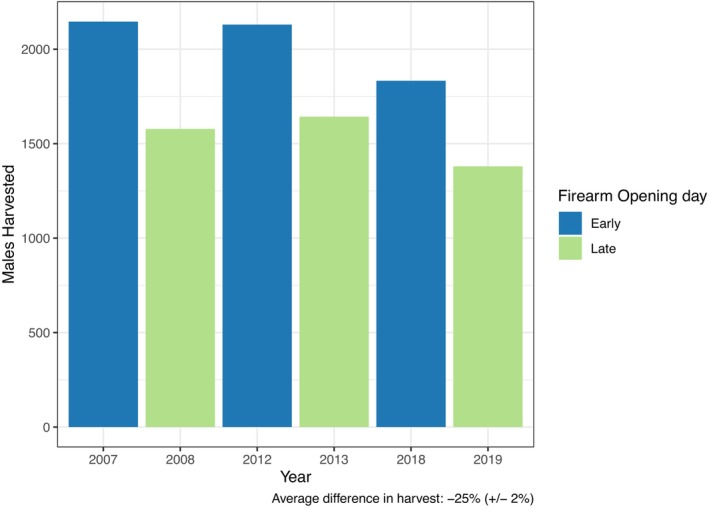
Number of male white‐tailed deer ( *Odocoileus virginianus* ) harvested in Iowa county Wisconsin (WI) using a firearm in pairs of years in which the earliest possible opening day for firearm season (November 17) is followed by the latest possible opening day for firearm season (November 23).

Movement rates showed no significant relationship with the hunting seasons or the timing of the opening weekend of the firearm season (Figure 3; Figure S3; Figure S4; Table S7). Both the opening weekend and the rest of the firearm season were associated with decreasing movement rates, but this pattern was better captured by the day number, as the decrease in movement rate started prior to the hunting season. However, a trend was observed for increased deer harvest in years when the opening day of firearm season began on November 17—closer to the peak rut, when movement rates are high—compared to years when opening day was November 23 (Figure 4; Figure S5). The mean movement rate (2017–2020) on November 23rd was 6% lower on average than on November 17th and 19% lower between November 23, 2019 and November 17, 2018. With the change in the opening day of the firearm season from November 17 in 2018 to November 23 in 2019, male harvest in Iowa County, Wisconsin declined 25% (Figure 4).

**FIGURE 5:**
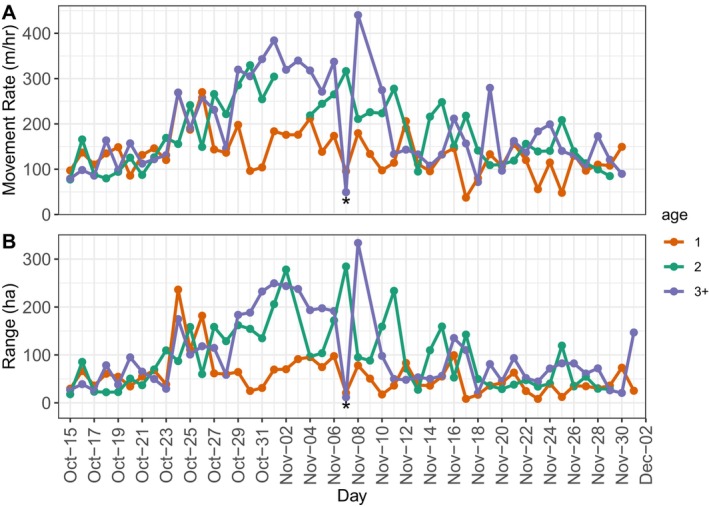
Average daily movement rates (m/h) (A) and range sizes (daily 95% Utilization Distribution) (B) by age (1, 2, 3+) of an individual male white‐tailed deer ( *Odocoileus virginianus* ) (ID 5700) over three breeding seasons (2017–2019) in southwest Wisconsin. The * on November 07 indicates a potential breeding event as a 3‐year‐old with a low movement rate and small daily range.

In the top individual‐level model, the residual movement rate variance was higher ($\varepsilon_{\mathrm{ij}}$ = 6.72) than that for individual deer ($A_{i}$ = 2.53), indicating that within‐deer heterogeneity accounted for more of the variability in individual movement rates than did between‐deer heterogeneity (i.e., variance in individual deer movement rates is not generally the result of having some individuals with consistently high or low rates). However, while we lacked sufficient individuals monitored over multiple years for robust statistical analysis, some individuals did show evidence for changing their movement rate patterns with age. As an example, one male that aged from 1 to 3 years old had high variation in daily movement rates and ranges within a season, with interannual trends that were similar to those of the corresponding age‐level averages observed among individuals (Figure 2; Figure 5; Figure S6).

### 3.4 Daily Activity Patterns

**FIGURE 6:**
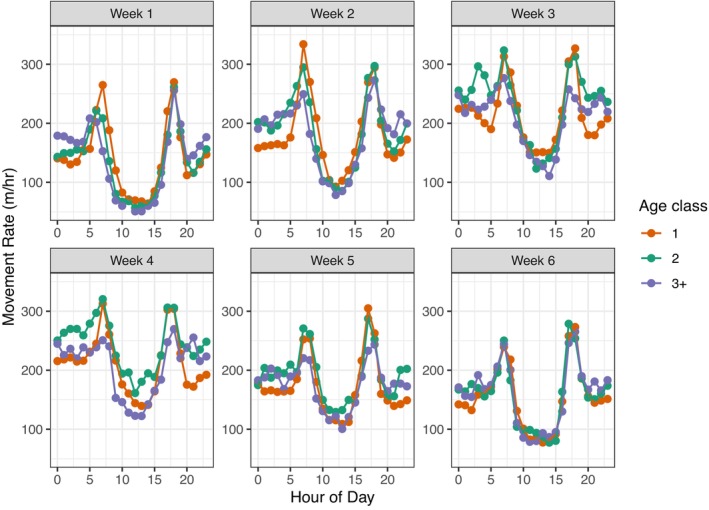
Activity patterns of male white‐tailed deer ( *Odocoileus virginianus* ) based on mean hourly movement rates (m/h) by week and age (1, 2, 3+ years) during the breeding season in southwest Wisconsin during 2017–2020. Week 1: October 15–October 21; Week 6: November 19–November 25. Peak activity occurred during Week 4 (November 5–November 11) based on mean movement rate of all ages.

When focusing on deer movement patterns within a day, we found that movement rates across ages generally increased across the 24‐h period to a mean maximum movement rate in the week of November 5–November 11 (week 4; Figure 6; Figure S7). During this period, the 3+ had lower movement rates at dawn compared to yearlings and 2‐year‐olds (pairwise comparison; *p* = 0.0001). Yearlings and 2‐year‐olds had slightly elevated movement rates during the day compared to 3+ (pairwise comparisons; *p* < 0.0001). At dusk, all ages had similar movement rates, while at night, both the 2‐year‐olds and 3+ had higher movement rates than yearlings (pairwise comparison; *p* < 0.01). The 3+ showed signs of moving from crepuscular activity patterns to transitioning towards nocturnal activity patterns during the peak rut (Figure 6). The linear regression describing movement rate related to age, week number, and period of day was fit with an adjusted *R* ^2^ of 0.96.

## 4 Discussion

We evaluated movement patterns of male deer during the rut to determine how reproductive strategies vary with age and how movement metrics can identify biologically relevant breeding periods. We found that age was a key driver of male movement rates and range sizes, as well as variance in these movement metrics. In addition, we found that change‐point analysis of movement rates and range sizes consistently identified late October through early November as the peak rut, and that this timing aligned with back dated parturition data. Our results describe male movement behavior during the rut by age and external factors. Additionally, we used male movement data and estimated conception dates to assess the timing of the rut. These findings may inform wildlife management decisions and guide future research during this key biological period.

The differences in movement behavior metrics we observed appear to be the result of age‐related changes in strategies for male deer searching for mates and their ability to successfully tend and defend females. For example, across the rut, we found that by age averages, 2‐year‐old males had higher daily movement rates and larger daily ranges than yearlings and 3+. This would be consistent with 2‐year‐olds using a wide‐ranging, Levy walk‐type strategy, as compared to yearlings. Varying reproductive strategies were also evident from within‐day activity patterns, with the yearlings and 3+ having similar movement rates, but divergence in the timing of their peak activity. In this case, yearlings were more active during dawn hours and 3+ were more active during the night, especially during the peak rut. The observed age differences in male ranging and activity during the rut could be driven by physiological or behavioral mechanisms. For example, yearlings may be investing more in survival or growth than reproduction until physical maturity (Yoccoz et al. 2002; Forsyth et al. 2005; Byers et al. 2010). Alternatively, due to inexperience and a lack of competitive ability (Sandell 1986; Koprowski 1993), yearlings may be using a more sedentary strategy (Kodric‐Brown 1986) in response to lower testosterone levels than more mature males (Gomes et al. 2021).

We found some evidence for within‐individual shifts in reproductive strategy with age (Figure 5; Figure S6), though we lacked sufficient multiyear observations of individual deer for a robust statistical analysis. As an example, activity data from a single individual shown in Figure 5 demonstrate a shift from the lower movement rates and smaller ranges typical of yearlings to the higher movement rates and variance in movement patterns associated with 2‐year olds and 3+. Thus, this individual likely transitioned from a more sedentary strategy as a yearling (Kodric‐Brown 1986) to a Levy walk or roving strategy as an older adult. Future work with longer‐term monitoring of individuals could be helpful to refine our understanding of the frequency of within‐individual changes in reproductive strategy and how such shifts might be affected by individual personality (Nilsson et al. 2014), local deer density (Lutz et al. 2015), or habitat (Gilbertson et al. 2022).

Shifts in reproductive strategy with age likely also result in changes to reproductive success. For example, a potential tending event is shown for an individual male at 3+ (Figure 5). This individual's movement rate and range size at 3+ generally increased during the peak rut, then abruptly dropped on November 7 in a presumptive tending event. Movement then peaked November 8, suggesting a return to a searching state (Figure 5). These types of changes in movement patterns during tending events mean that variance in male daily movement rates and ranges may be indicators of breeding success. We did not attempt to enumerate potential tending events but rather use this as an example to demonstrate the concept of searching and tending behavior that would lead to higher variance. We observed that male movement rate variance increased during peak rut for all ages, with the 3+ showing the highest variance during the peak rut, followed by 2‐year olds and yearlings, respectively. In addition, the smaller ranges we observed for the 3+, coupled with equivalent range size variance to 2‐year olds, suggest that older individuals were more often switching from likely searching behavior (i.e., large daily ranges) to tending behavior (i.e., small daily ranges; Figure 2).

The higher movement rates and range variance we observed among 2‐year‐olds and 3+ would support the notion that these individuals typically have the highest breeding success. Indeed, this aligns with previous studies which found that yearlings are typically less successful at breeding than older males (Sorin 2004; DeYoung et al. 2009; Turner et al. 2016). Alternatively, the higher movement rate variance among 2‐year‐olds and 3+ males could be attributable to heterogeneity between individuals, with some individuals having very high or very low movement rates, but no actual within‐individual behavioral changes. However, variance partitioning in our mixed model for movement rates suggests this is not the case. Rather, the observed high within‐individual heterogeneity in movement rates suggests that most individuals are indeed switching from high to low movement rate states. A tending event, however, does not necessarily mean a successful breeding event. For example, while tending a female, if the tending male is actively defending against other males, one may have the opportunity to breed with the female while the tending male is preoccupied with another male (Isvaran 2005). Future directions may include genetic‐based paternity in enclosed populations and fine‐scale movement data to pair breeding success by age and movement behaviors.

Changepoint analysis of mean daily movement rate and range size during the rut, or of estimated neonate conception dates may be a useful tool for identifying peak breeding activity and defining ecologically relevant study periods for future research. Previous studies have characterized the entire breeding season, spanning multiple months for varied purposes such as survival, dispersal, and migration without the aid of GPS technologies (Hölzenbein and Schwede 1989; Nixon et al. 1991; Skuldt et al. 2008). In contrast, in temperate populations with balanced sex ratios and age structure, the majority of females are bred in a 2–4 week period (DeYoung and Miller 2011; Hewitt 2011). Here, changepoint analysis of daily movement metric averages (movement rate and range size) identified peak rut length of approximately 3 weeks and was generally supported by estimated fawn neonate conception dates. It may be expected that peak rut, based on conception dates, would be narrower compared to male movement metrics as male movement metrics may begin to increase sharply as the first females come into estrus and subsequently steadily decline as fewer females come back into estrus a second time after the peak. Nevertheless, both male movement patterns and backdating from fawn birth dates are useful tools and show agreement in defining the breeding periods for deer.

From a wildlife management perspective, the timing of the rut is particularly important in the context of the recreational hunting season. Increases in movement related to the rut may make deer more vulnerable to harvest (Little et al. 2014), but high hunting pressure may simultaneously alter deer movement patterns (Frid and Dill 2002; Little et al. 2016). Thus, when high hunting pressure and the rut occur concurrently, it can be difficult to identify which process is driving changes in movement behavior. We found no effect of the firearm hunting season, including opening weekend, on deer movement rates. Our findings contrast with previous work which found that males decreased movement during firearm hunting season (Little et al. 2016; Karns et al. 2012); however, the latter study occurred post‐rut and at low hunting pressure. The lack of an effect of hunting season on movement rates in our study could be due to variability in hunting pressure across our study area, such that individuals may have either increased or decreased movement, depending on the local hunting pressure. Alternatively, rut‐induced changes in movement behavior may predominate over or otherwise obscure hunting‐induced changes in our region.

Furthermore, rut‐related behaviors appear to affect hunter harvest. While year‐to‐year changes in male harvest may be due to many factors (Webb et al. 2010; Little et al. 2014), our results suggest that the timing of the recreational hunting season relative to the rut can play a role in shaping harvest success. The relationship between the timing of harvest and the rut is especially important in the context of infectious disease management, where harvest is often the primary means of disease control (Heberlein 2004; Uehlinger et al. 2016).

In an infectious disease context, male movement and contact patterns during the rut are thought to influence disease transmission dynamics and spatial spread (Samuel and Storm 2016). In the case of CWD, prevalence in cervid species is higher in older age classes and can be two times higher in adult males than females (Ketz et al. 2019). These patterns are thought to result, at least in part, from male breeding behaviors (Samuel and Storm 2016; Gilbertson et al. 2025). Our findings suggest that older males, with higher presumed breeding success, may pose a high risk of transmission to females. Additionally, while yearlings may contribute to spatial spread through dispersal (Skuldt et al. 2008; Gilbertson et al. 2022), 2‐year‐olds had the largest ranges during peak rut, suggesting they may also play a role in driving CWD spatial spread. Future research could examine how individual behaviors influence the risk of acquiring and transmitting CWD. In addition to searching and breeding, this could include other potentially risky behaviors such as social grooming, sparring and fighting, and the use of scrapes, the latter of which are thought to be an important point of CWD transmission risk (Kinsell 2010; Egan et al. 2023).

In this study, we quantified the timing of the peak rut and the associated movement behavior in male deer. Our results suggest that as an individual ages, they alter their movement behaviors related to mate search and breeding success. These findings agree with and build on the body of cervid breeding season literature and provide key information for deer population management and disease control.

## Author Contributions

**Matthew A. Hunsaker:** conceptualization (lead), data curation (equal), formal analysis (lead), investigation (lead), methodology (equal), project administration (supporting), validation (supporting), visualization (lead), writing – original draft (lead), writing – review and editing (lead). **Marie L. J. Gilbertson:** conceptualization (supporting), data curation (equal), formal analysis (supporting), investigation (supporting), methodology (supporting), project administration (supporting), supervision (supporting), validation (lead), visualization (supporting), writing – original draft (supporting), writing – review and editing (supporting). **Daniel J. Storm:** conceptualization (supporting), funding acquisition (equal), investigation (supporting), methodology (supporting), project administration (equal), resources (lead), supervision (supporting), writing – original draft (supporting), writing – review and editing (supporting). **Wendy C. Turner:** conceptualization (supporting), funding acquisition (equal), investigation (supporting), methodology (supporting), project administration (lead), resources (supporting), supervision (lead), writing – original draft (supporting), writing – review and editing (supporting).

## Conflicts of Interest

The authors declare no conflicts of interest.

## Supporting information


Appendix S1.

## Data Availability

At the time of publication, data were not available from the Wisconsin Department of Natural Resources; restrictions apply to the availability of these data, which were used under a data sharing agreement for the current study and so are not publicly available. Data may be requested from the Wisconsin Department of Natural Resources through Daniel Storm (danielj.storm@wisconsin.gov).
