# Supplementary material for: The Breeding Season and Movement Ecology of Male White‐Tailed Deer in Southwest Wisconsin
Source: Ecol Evol. 2025 Jul 9;15(7):e71589. doi: 10.1002/ece3.71589 (PMC12240682; doi:10.1002/ece3.71589)
Supplement: Supplementary file 1 — Appendix S1. [file ECE3-15-e71589-s001.docx]

**Supplementary materials for “The breeding season and movement ecology of male white-tailed deer in southwest Wisconsin”**

Matthew A. Hunsaker^1^*, Marie L. J. Gilbertson^1^, Daniel J. Storm^2^, Wendy C. Turner^3^

^1^Wisconsin Cooperative Wildlife Research Unit, Department of Forest and Wildlife Ecology, University of Wisconsin–Madison, 1630 Linden Dr., Madison, Wisconsin, 53706, USA

^1^[mahunsaker95@gmail.com](mailto:mahunsaker95@gmail.com); [mjones029@gmail.com](mailto:mjones029@gmail.com)

^2^Wisconsin Department of Natural Resources, 1300 West Clairemont Ave., Eau Claire, WI 54701

^2^DanielJ.Storm@wisconsin.gov

^3^U.S. Geological Survey, Wisconsin Cooperative Wildlife Research Unit, Department of Forest and Wildlife Ecology, University of Wisconsin–Madison, 1630 Linden Dr., Madison, Wisconsin, 53706, USA

[^3^wendy.turner@wisc.ed](mailto:3wendy.turner@wisc.edu)u

*Corresponding author: [mahunsaker95@gmail.com](mailto:mahunsaker95@gmail.com)

**Any use of trade, product, or firm names is for descriptive purposes only and does not imply endorsement by the U.S. Government.**

**MATERIALS AND METHODS**

**Table S1.**—Yearly summaries of global positioning system (GPS) collared male white-tailed deer (*Odocoileus virginianus*) sample size, average age (years), average fix rate (fixes per hour), and the opening day of the Wisconsin Department of Natural Resources firearm hunting season.

| Year | Sample size | Average age (years) | Average fix rate | Opening day (firearm) |
| --- | --- | --- | --- | --- |
| 2017 | 39 | 1.49 | 1.02 | Nov 18 |
| 2018 | 54 | 1.87 | 1.02 | Nov 17 |
| 2019 | 65 | 1.83 | 1.02 | Nov 23 |
| 2020 | 74 | 1.97 | 1.02 | Nov 21 |

**Polynomial regression equations**

Here we show the top polynomial regression models used in predicting daily mean movement rate (in m/h; $m$; Equation 1), daily mean range size (in ha; $r$; Equation 2), log daily movement rate variance (Equation 3), and log daily range variance (Equation 4).

Equation 1.

$$m_{ij} = \beta_{0}+\beta_{1}d_{1ij}+\beta_{2}{d^{2}}_{2ij}+\beta_{3}{d^{3}}_{3ij}+\beta_{4}{d^{4}}_{4ij}+\beta_{5}{{d^{5}}_{5ij}}+\beta_{6}{d^{6}}_{6ij}+\beta_{7}a_{1ij}+\beta_{8}(ad)_{1ij}+\beta_{9}(ad{)^{2}}_{2ij}+\beta_{10}(ad{)^{3}}_{3ij}+\beta_{11}(a{{d)}^{4}}_{4ij} + \varepsilon$$

Equation 2.

$$r_{ij} = \beta_{0}+\beta_{1}d_{1ij}+\beta_{2}{d^{2}}_{2ij}+\beta_{3}{d^{3}}_{3ij}+\beta_{4}{d^{4}}_{4ij}+\beta_{5}{{d^{5}}_{5ij}}+\beta_{6}a_{1ij}+\beta_{7}(ad)_{1ij}+\beta_{8}(ad{)^{2}}_{2ij} + \varepsilon$$

Equation 3.

$${log(var(r}_{ij})) = \beta_{0}+\beta_{1}d_{1ij}+\beta_{2}{d^{2}}_{2ij}+\beta_{3}{d^{3}}_{3ij}+\beta_{4}{d^{4}}_{4ij}+\beta_{5}a_{1ij}+\beta_{6}(ad)_{1ij}+\beta_{7}(ad{)^{2}}_{2ij} + \varepsilon$$

Equation 4.

$${log(var(m}_{ij})) = \beta_{0}+\beta_{1}d_{1ij}+\beta_{2}{d^{2}}_{2ij}+\beta_{3}{d^{3}}_{3ij}+\beta_{4}{d^{4}}_{4ij}+\beta_{5}a_{1ij}+\beta_{6}(ad)_{1ij}+\beta_{7}(ad{)^{2}}_{2ij} + \varepsilon$$

**RESULTS**

**
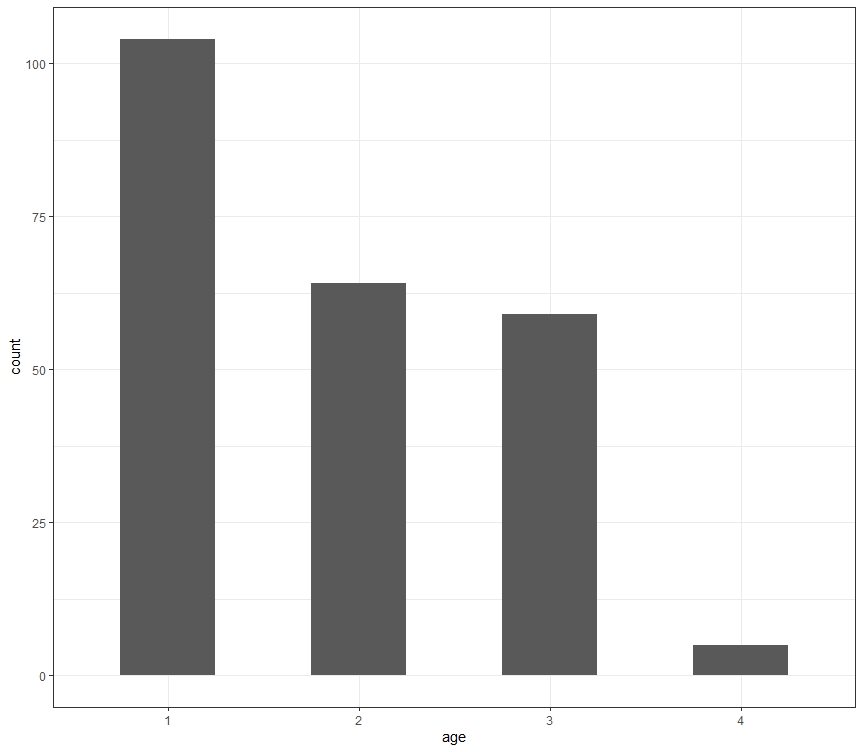
**

**Fig. S1.**—Number of individual male white-tailed deer (*Odocoileus virginianus*) per age that had sufficient GPS collar data for data analysis from southwest Wisconsin during 2017-2020.

**Table S2.**—Start and end dates and length of peak rut for white-tailed deer (*Odocoileus virginianus*) based on changepoint analysis of movement rate and range size of all sampled males, by age class of males, and from estimated conception dates based on neonate capture data.

| Data | Start | End | Length |
| --- | --- | --- | --- |
| Conception dates | October 27 | November 11 | 16 days |
| 1 year old movement rate | October 24 | November 12 | 20 days |
| 2 year old movement rate | October 23 | November 15 | 24 days |
| 3+ year old movement rate | October 23 | November 12 | 21 days |
| 1 year old range | October 23 | November 12 | 21 days |
| 2 year old range | October 23 | November 15 | 24 days |
| 3+ year old range | October 23 | November 9 | 18 days |
| All male range | October 23 | November 11 | 20 days |
| All male movement rate | October 23 | November 12 | 21 days |

**Table S3. .**—Model results for polynomial regression model of movement rate of male white-tailed deer (*Odocoileus virginianus*) in southwestern Wisconsin. 95% confidence intervals (CI) are shown. Statistically significant results are highlighted with bold text. The day^x^ parameter describes the day number covariate raised to the power of x. Std. Error = standard error; 95% CI = 95% confidence interval.

|  | **Model term** | **Exp(estimate)** | **Std. Error** | **95% CI** |
| --- | --- | --- | --- | --- |
| movement rate (m/hr) model | **Intercept** | **174.45** | **1.81** | **170.88 – 178.02** |
|  | **Day** | **-56.61** | **21.67** | **-99.47 – -13.74** |
|  | **Day^2^** | **-292.02** | **21.67** | **-334.89 – -249.16** |
|  | **Day^3^** | **126.79** | **21.67** | **83.92 – 169.65** |
|  | **Day^4^** | **99.11** | **21.67** | **56.24 – 141.97** |
|  | **Day^5^** | **-71.47** | **12.51** | **-96.22 – -46.72** |
|  | **Day^6^** | **-25.14** | **12.51** | **-49.88 – -0.39** |
|  | **2-year old age class** | **11.16** | **2.56** | **6.11 – 16.21** |
|  | 3+ year old age class | 0.61 | 2.56 | -4.44 – 5.66 |
|  | 2-year old age class:Day | 21.61 | 30.64 | -39.02 – 82.23 |
|  | 3+ year old age class:Day | 56.32 | 30.64 | -4.30 – 116.94 |
|  | **2-year old age class:Day^2^** | **-140.25** | **30.64** | **-200.87 – -79.63** |
|  | 3+ year old age class:Day^2^ | 17.94 | 30.64 | -42.68 – 78.57 |
|  | 2-year old age class:Day^3^ | 25.48 | 30.64 | -35.15 – 86.10 |
|  | 3+ year old age class:Day^3^ | 1.73 | 30.64 | -58.89 – 62.35 |
|  | 2-year old age class:Day^4^ | 43.02 | 30.64 | -17.61 – 103.64 |
|  | 3+ year old age class:Day^4^ | -52.66 | 30.64 | -113.29 – 7.96 |

|  | **Model term** | **Exp(estimate)** | **Std. Error** | **95% CI** |
| --- | --- | --- | --- | --- |
| Range (ha) model | **Intercept** | **83.54** | **1.39** | **80.80 – 86.29** |
|  | **Day** | **-41.82** | **16.85** | **-75.14 – -8.50** |
|  | **Day^2^** | **-161.72** | **16.85** | **-195.04 – -128.40** |
|  | **Day^3^** | **104.92** | **9.73** | **85.68 – 124.15** |
|  | **Day^4^** | **51.29** | **9.73** | **32.06 – 70.53** |
|  | **Day^5^** | **-30.38** | **9.73** | **-49.62 – -11.15** |
|  | **2-year old age class** | **8.78** | **1.97** | **4.90 – 12.67** |
|  | 3+ year old age class | 2.13 | 1.97 | -1.76 – 6.01 |
|  | 2-year old age class:Day | 25.47 | 23.83 | -21.65 – 72.59 |
|  | **3+ year old age class:Day** | **89.03** | **23.83** | **41.91 – 136.15** |
|  | **2-year old age class:Day^2^** | **-81.81** | **23.83** | **-128.93 – -34.69** |
|  | 3+ year old age class:Day^2^ | 41.95 | 23.83 | -5.17 – 89.07 |

**Table S4. .**—Model results for polynomial regression model of daily range (95% utilization distribution) of male white-tailed deer (*Odocoileus virginianus*) in southwestern Wisconsin. 95% confidence intervals (CI) are shown. Statistically significant results are highlighted with bold text. The day^x^ parameter describes the day number covariate raised to the power of x. Std. Error = standard error; 95% CI = 95% confidence interval.

**Table S****5.**—Model results for polynomial regression model of Log variance of movement rate of male white-tailed deer (*Odocoileus virginianus*) in southwestern Wisconsin. 95% confidence intervals (CI) are shown. Statistically significant results are highlighted with bold text. The day^x^ parameter describes the day number covariate raised to the power of x. Std. Error = standard error; 95% CI = 95% confidence interval.

|  | **Model term** | **Exp(estimate)** | **Std. Error** | **95% CI** |
| --- | --- | --- | --- | --- |
| Log(Variance movement rate (m/hr)) model | **Intercept** | **8.39** | **0.03** | **8.33 – 8.45** |
|  | Day | -0.20 | 0.36 | -0.92 – 0.52 |
|  | **Day^2^** | **-2.89** | **0.36** | **-3.60 – -2.17** |
|  | **Day^3^** | **1.30** | **0.21** | **0.89 – 1.72** |
|  | **Day^4^** | **1.04** | **0.21** | **0.63 – 1.46** |
|  | **2-year old age class** | **0.22** | **0.04** | **0.13 – 0.30** |
|  | **3+ year old age class** | **0.39** | **0.04** | **0.31 – 0.47** |
|  | **2-year old age class:Day** | **2.06** | **0.51** | **1.05 – 3.08** |
|  | 3+ year old age class:Day | -0.41 | 0.51 | -1.43 – 0.60 |
|  | **2-year old age class:Day^2^** | **-3.63** | **0.51** | **-4.64 – -2.61** |
|  | **3+ year old age class:Day^2^** | **-3.35** | **0.51** | **-4.37 – -2.33** |

**Table S6.**—Model results for polynomial regression model of Log variance of daily range (95% utilization distribution) of male white-tailed deer (*Odocoileus virginianus*) in southwestern Wisconsin. 95% confidence intervals (CI) are shown. Statistically significant results are highlighted with bold text. The day^x^ parameter describes the day number covariate raised to the power of x. Std. Error = standard error; 95% CI = 95% confidence interval.

|  | **Model term** | **Exp(estimate)** | **Std. Error** | **95% CI** |
| --- | --- | --- | --- | --- |
| Log(variance Range (ha)) model | **Intercept** | **8.14** | **0.05** | **8.05 – 8.24** |
|  | **Day** | **-1.93** | **0.60** | **-3.11 – -0.75** |
|  | **Day^2^** | **-2.81** | **0.60** | **-3.99 – -1.63** |
|  | **Day^3^** | **1.96** | **0.34** | **1.28 – 2.64** |
|  | Day^4^ | 0.62 | 0.34 | -0.06 – 1.30 |
|  | 2-year old age class | 0.02 | 0.07 | -0.12 – 0.15 |
|  | 3+ year old age class | 0.08 | 0.07 | -0.06 – 0.21 |
|  | **2-year old age class:Day** | **3.34** | **0.84** | **1.67 – 5.00** |
|  | **3+ year old age class:Day** | **3.29** | 0.84 | **1.62 – 4.96** |
|  | **2-year old age class:Day^2^** | **-3.24** | 0.84 | **-4.91 – -1.58** |
|  | 3+ year old age class:Day^2^ | -1.64 | 0.84 | -3.31 – 0.03 |

**Table S7.**—Model results for polynomial mixed model of square root transformed movement rate of male white-tailed deer (*Odocoileus virginianus*) in southwestern Wisconsin. 95% confidence intervals (CI) are shown. Statistically significant results are highlighted with bold text. The day^x^ parameter describes the day number covariate raised to the power of x. Std. Error = standard error; 95% CI = 95% confidence interval.

|  | **Model term** | **Exp(estimate)** | **Std. Error** | **95% CI** |
| --- | --- | --- | --- | --- |
| sqrt(movement rate (m/hr) model | **Intercept** | **13.15** | **0.19** | **12.78 – 13.52** |
|  | Day | -19.08 | 13.70 | -45.94 – 7.77 |
|  | **Day^2^** | **-86.64** | **12.91** | **-111.94 – -61.34** |
|  | **Day^3^** | **31.60** | **8.68** | **14.57 – 48.62** |
|  | **Day^4^** | **26.56** | **4.63** | **17.50 – 35.63** |
|  | **Day^5^** | **-17.47** | **2.97** | **-23.29 – -11.65** |
|  | Day^6^ | -4.55 | 3.39 | -11.20 – 2.09 |
|  | **2-year old age class** | **0.43** | **0.11** | **0.21 – 0.65** |
|  | **3+ year old age class** | **-0.34** | **0.17** | **-0.68 – 0.00** |
|  | Change in temperature | -0.01 | 0.005 | -0.02 – 0.00 |
|  | Opening weekend of firearm hunting season | -2.40 | 2.78 | -7.84 – 3.05 |
|  | Firearm hunting season (not including opening weekend) | -2.11 | 3.47 | -8.91 – 4.69 |
|  | 2-year old age class:Day | 0.84 | 6.53 | -11.95 – 13.63 |
|  | 3+ year old age class:Day | 6.57 | 7.43 | -7.99 – 21.12 |
|  | **2-year old age class:Day^2^** | **-30.79** | **6.29** | **-43.12 – -18.46** |
|  | 3+ year old age class:Day^2^ | 9.27 | 6.96 | -4.38 – 22.91 |
|  | Opening weekend of firearm hunting season:Day | 0.08 | 0.08 | -0.07 – 0.23 |
|  | Firearm hunting season (not including opening weekend):Day | 0.05 | 0.09 | -0.13 – 0.23 |

**
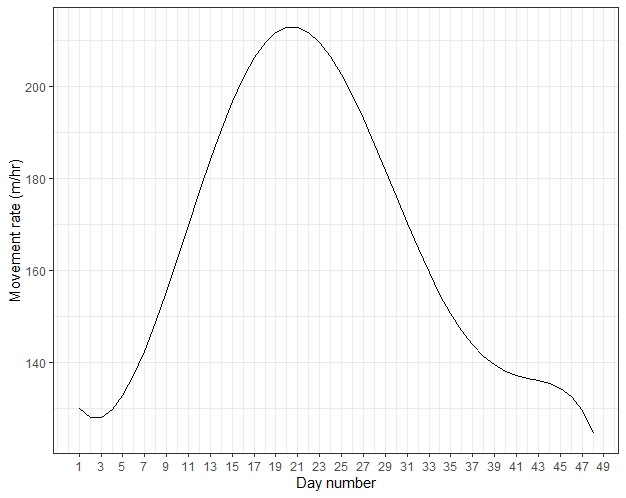
**

**Fig. S2.**—Effects plot of day number from model results for polynomial mixed model of square root transformed movement rate of male white-tailed deer (*Odocoileus virginianus*) in southwestern Wisconsin. Day number was fit to a 6th order polynomial.


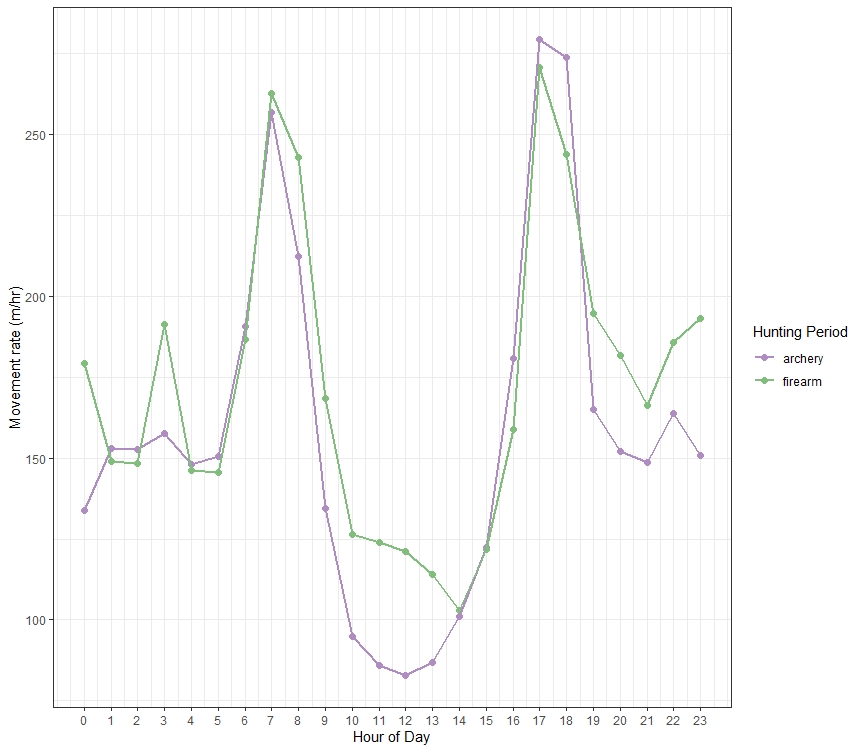


**Fig. S3.**—Activity patterns of male white-tailed deer (*Odocoileus virginianus*) based on mean hourly movement rates (m/hr) by hunting period in southwest Wisconsin during 2017-2020. The archery hunting period is the two days prior to the major firearm hunting season and the firearm hunting period is the first two days of the major firearm season.


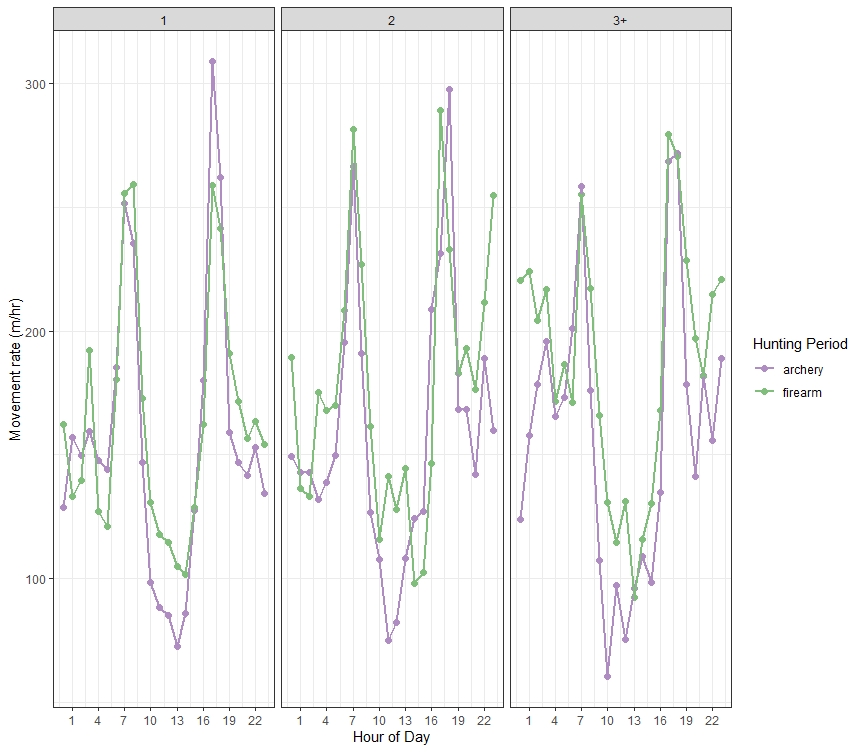


**Fig. S4.**—Activity patterns of male white-tailed deer (*Odocoileus virginianus*) based on mean hourly movement rates (m/hr) by hunting period and age class in southwest Wisconsin during 2017-2020. The archery hunting period is the two days prior to the major firearm hunting season and the firearm hunting period is the first two days of the major firearm season.


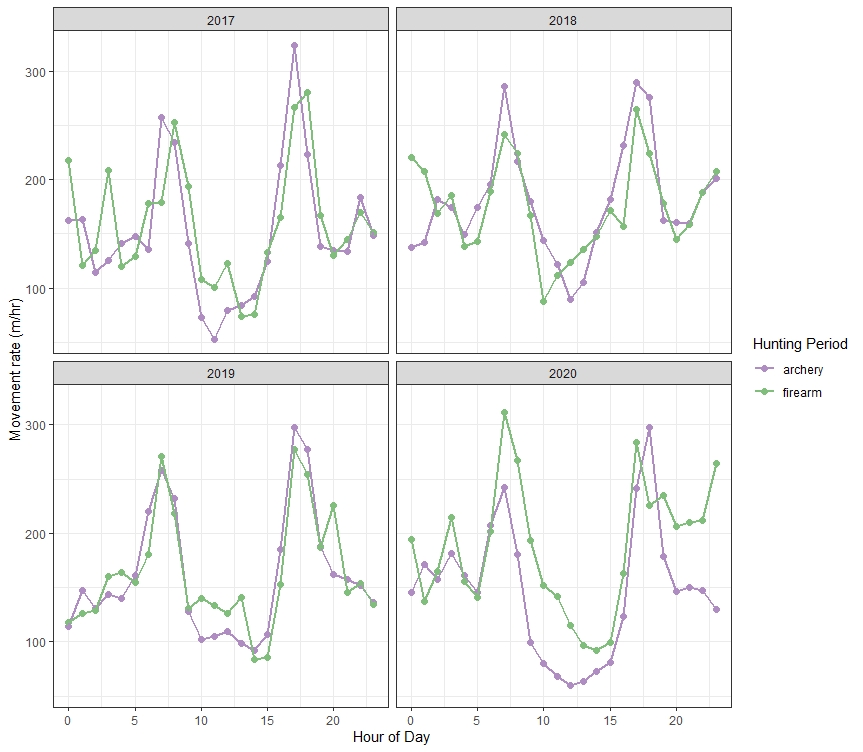


**Fig. S5.**—Activity patterns of male white-tailed deer (*Odocoileus virginianus*) based on mean hourly movement rates (m/hr) by hunting period and year in southwest Wisconsin during 2017-2020. The archery hunting period is the two days prior to the major firearm hunting season and the firearm hunting period is the first two days of the major firearm season.


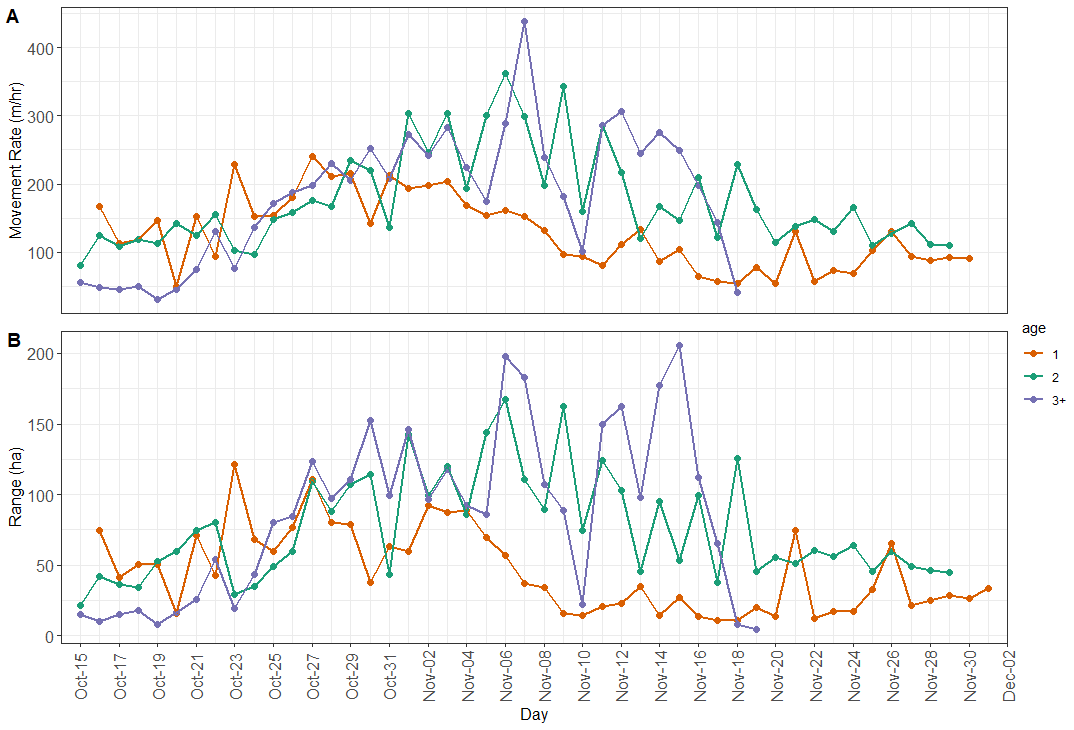


**Fig. S6.**—Average daily movement rates (m/hr) (A) and ranges (95% Utilization Distribution) (B) by age class (1, 2, 3+) of an individual male white-tailed deer (*Odocoileus virginianus*) (ID 5710) over 3 breeding seasons (2017-2019) in southwest Wisconsin.

**
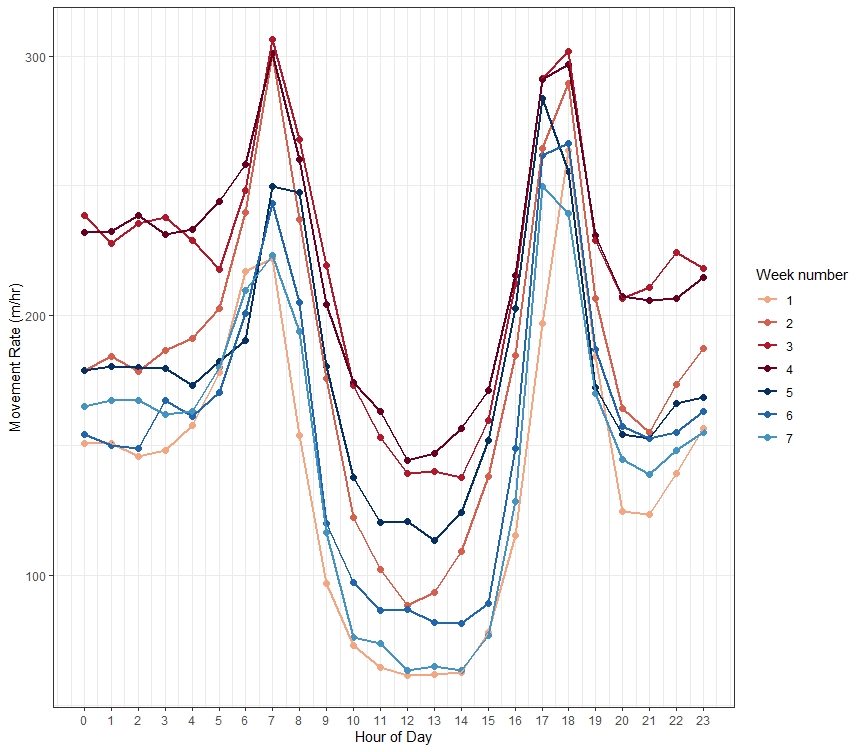
**

**Fig. S7.**—Activity patterns of male white-tailed deer (*Odocoileus virginianus*) based on mean hourly movement rates (m/hr) by week during the breeding season in southwest Wisconsin during 2017-2020. (Week 1 = Oct 15 - Oct 21)
